# A Cascade of Interventions to Promote Adherence to Antiretroviral Therapy in African Countries

**DOI:** 10.1007/s11904-020-00511-4

**Published:** 2020-08-10

**Authors:** Rebecca Jopling, Primrose Nyamayaro, Lena S Andersen, Ashraf Kagee, Jessica E Haberer, Melanie Amna Abas

**Affiliations:** 1 Health Service & Population Research Department, Institute of Psychiatry, Psychology & Neuroscience, King’s College London, London, UK; 2 Department of Psychiatry, University of Zimbabwe College of Health Sciences, Mazowe Street, Avondale, Harare, Zimbabwe; 3 HIV Mental Health Research Unit, Division of Neuropsychiatry, Department of Psychiatry and Mental Health, University of Cape Town, Groote Schuur Hospital Anzio Road, Observatory, Cape Town, South Africa; 4 Department of Psychology, Stellenbosch University, Stellenbosch, 7602 South Africa; 5 Center for Global Health, Massachusetts General Hospital, Harvard Medical School, Boston, USA

**Keywords:** HIV, Interventions, Adherence, Antiretroviral therapy (ART), Retention in care, ART initiation, Review

## Abstract

**Purpose of Review:**

We reviewed interventions to improve uptake and adherence to antiretroviral therapy (ART) in African countries in the Treat All era.

**Recent Findings:**

ART initiation can be improved by facilitated rapid receipt of first prescription, including community-based linkage and point-of-care strategies, integration of HIV care into antenatal care and peer support for adolescents. For people living with HIV (PLHIV) on ART, scheduled SMS reminders, ongoing intensive counselling for those with viral non-suppression and economic incentives for the most deprived show promise. Adherence clubs should be promoted, being no less effective than facility-based care for stable patients. Tracing those lost to follow-up should be targeted to those who can be seen face-to-face by a peer worker.

**Summary:**

Investment is needed to promote linkage to initiating ART and for differentiated approaches to counselling for youth and for those with identified suboptimal adherence. More evidence from within Africa is needed on cost-effective strategies to identify and support PLHIV at an increased risk of non-adherence across the treatment cascade.

**Electronic supplementary material:**

The online version of this article (10.1007/s11904-020-00511-4) contains supplementary material, which is available to authorized users.

## Introduction

The establishment of the 90-90-90 goals was a turning point for African countries, with the potential to save millions of lives and avert future costs for patients and their families, the healthcare system and national economies. However, at population level, only 58% of people living with HIV (PLHIV) in Eastern and Southern Africa and 39% in West and Central Africa were virally supressed in 2018 [1], suggesting a high need for improved adherence support, as well as greater advances in testing. There are national variations across these regions, with progress in some African countries alarmingly slow and considerable data missing on viral suppression in over 20 countries [1]. Progress towards the UNAIDS goals has been particularly slow among key populations, i.e. gay men and other men who have sex with men, sex workers, transgender people, people who inject drugs and prisoners.

The UNAIDS 90-90-90 goals map the HIV care cascade in terms of knowledge of HIV status, sustained receipt of antiretroviral therapy (ART) and attainment of viral suppression [2]. The second and third ‘90s’ are highly dependent on adherence [3, 4]. Adherence to ART involves a number of components along the HIV care cascade, namely linking with care to initiate treatment, the need for ongoing engagement (e.g. regular clinic attendance), taking ART as prescribed and ensuring that therapy persists without interruption or discontinuation [5].

Numerous developments in ART regimens and treatment policies in the past several years have had major influence on the need and manner in which adherence should be supported for treatment success. For instance, the availability of safer and more efficacious ART regimens has increased as integrase inhibitors have become more affordable for low- and middle-income countries (LMICs). Evidence that immediate initiation of ART after a positive HIV test result could contribute to epidemic control prompted the launch of the World Health Organization’s Treat All guidelines in 2016 [6]. This strategy has brought about considerable changes in many African countries, with Test and Treat policies implemented in almost all countries [7].

Prior to Treat All, 23% of people on ART in sub-Saharan Africa were thought to have poor adherence [8]. With expansion of treatment in the Treat All era, barriers to adherence remain at multiple levels. Key barriers to adherence in African countries at a structural and health service level include the cost and availability of transportation, distance to clinic, oversubscribed healthcare services and drug stock-out [9]. At the individual level, barriers to adherence include beliefs about medicines, lack of motivation, side effects, lack of access to adequate food, stigma, lack of social support and poor relationships with healthcare providers [9, 10]. Common mental disorders, such as depression, are both highly prevalent in people living with HIV and have consistently been associated with worse adherence to ART in LMICs [11, 12]. Key barriers to adherence can change over time, shaped by life events and changes in circumstances.

Interventions to promote adherence to ART encompass strategies across the HIV care cascade. It is unlikely that a one-size-fits-all approach is applicable, and careful consideration should be given to understand in which settings interventions work best and what interventions are effective for high-risk groups including youth, pregnant women, those with comorbid mental disorders and key populations.

This scoping review discusses strategies to improve adherence for five target populations along five components of the HIV treatment cascade: (1) PLHIV who know their status and are not yet initiated on ART, (2) all PLHIV prescribed ART, (3) ART users with sub-optimal adherence, (4) ART users with stable adherence and (5) PLHIV initiated on treatment who disengage from care for periods of time or are lost to follow-up (LTFU). Within these categories, we make a special note of populations facing unique challenges, such as youth, pregnant women and those with common mental disorders.

## Methods

Two separate searches were conducted to identify interventions for PLHIV across the care cascade. The first search identified interventions to increase ART initiation for PLHIV with known status not yet started on ART. The second identified interventions for PLHIV who had initiated treatment. Searches were limited to papers published from 1 January 2014 to 31 December 2019 to capture the era of Treat All in sub-Saharan Africa. Studies were excluded at full-text review if the study period ended before 2014 or was conducted before the Treat All policy era. For instance, we excluded studies of pregnant women prior to ‘Option B+’ [13].

### Search 1

We searched PubMed, EMBASE, Cochrane Central Register of Controlled Trials and Web of Science to identify relevant interventions for linkage in care specifically targeting initiation of ART or retention in care for those ART naive. To do this, we re-ran search terms from a recent systematic review of interventions to improve linkage to HIV care in the era of Treat All in sub-Saharan Africa [14]. The search terms for each database are described in the original article and included in Electronic Supplementary Material (ESM) 1. We updated the search to include studies from 1 January 2014 to 31 December 2019 and, in contrast to the original review, did not exclude studies focusing exclusively on pregnant women. See Table 1 for inclusion and exclusion criteria.

**Table 1:** Inclusion criteria for studies of antiretroviral therapy (ART) adherence interventions in the era of Treat All in sub-Saharan African nations (World Bank classification 2019) for each of five target populations along the HIV care cascade

	(1) PLHIV, who know their status and are not yet initiated on ART	(2) All PLHIV prescribed ART	(3) ART users with sub-optimal adherence	(4) ART users with stable adherence	(5) PLHIV initiated on treatment who disengage from care or are LTFU
Population	Known HIV+	Known HIV+ and initiated on ART	Known HIV+ and initiated on ART	Known HIV+ and initiated on ART	Known HIV+ and initiated on ART
Study type	Intervention to increase uptake/initiation of ART for those ART naïve	Intervention to promote adherence to ART	Intervention to promote adherence to ART for PLHIV needing adherence support	Intervention to promote adherence to ART for PLHIV considered ‘stable’ on treatment	Intervention to promote continued retention in care or re-engagement in care for those disengaged from care or lost to follow-up
Endpoint(s)^	Reported ART initiation^^	Viral suppression or ART adherence*	Viral suppression or ART adherence*	Viral suppression or ART adherence*	Retention or re-engagement in care, or viral suppression or ART adherence*
Exclusion criteria	- HIV testing only - Study population: only children aged ≤ 15 and studies involving mostly caregivers of PLHIV - RCTs of pharmacokinetics or drug trials	- Study population only children aged ≤ 15 and studies involving mostly caregivers of PLHIV - RCTs of pharmaco-kinetics or drug trials			

*RCT* randomised controlled trial

^Endpoints may have been primary or secondary

^^Typically measured using self-report or clinic records

*Measured by self-report, ART levels in blood, electronic medication monitoring devices and pharmacy ART refill data

A total of 5539 records were identified through database searches, and 1602 duplicates were removed. The remaining 3937 titles/abstracts were reviewed against the inclusion/exclusion criteria (see Table 1 below). Ultimately, 98 records were included for full-text review of which 31 studies were included of interventions for PLHIV not yet initiated on ART.

### Search 2

We ran a second search in PubMed, EMBASE, Cochrane Central Register of Controlled Trials and Web of Science to identify interventions for PLHIV who had been initiated on ART. The search terms for each database are described included in ESM 1. A total of 4906 records were identified through database searches, and 1746 duplicates were removed. The remaining 3160 titles/abstracts were reviewed against the inclusion criteria, and 190 records were included for full-text review. Twenty studies were included of interventions for PLHIV who are initiated on treatment. We divided these into four target populations across the HIV care cascade as shown in Table 1.

### Overview

This scoping review identified 51 articles. A total of 27 were randomised controlled trials (cluster or individually randomised), and 24 were non-randomised controlled trials.

### Target Population: PLHIV Who Know Their Status and Are Not Yet Initiated on ART

#### General Adult Populations

In the era of Test and Treat All, numerous approaches have been developed to increase ART initiation in general adult populations. These can be broadly divided into ways to initiate ART through home-based or community-based services, ways to support PLHIV to get to facilities and complex health systems strengthening interventions including integration of services. Many of the interventions tested have been enhanced through peer support and/or by technology (such as point-of-care tests and SMS reminders) and some through use of financial incentives.

Several studies have evaluated packages of home- or community-based services to identify PLHIV and initiate them on ART. In Lesotho, home-based HIV testing identified PLHIV who were then offered immediate ART initiation, with ongoing HIV care at a treatment facility [15^••^]. Home-based testing with immediate ART initiation increased linkage to care at a healthcare facility when compared to usual care (referral to the nearest health facility for preparatory counselling followed by ART initiation) (69% versus 43%; *p* = < 0.001) at 3 months; viral suppression as a marker of ART uptake and ongoing adherence was also higher at 12 months. A linkage to care cohort within the SEARCH trial in Kenya and Uganda offered HIV and multi-disease testing at health fairs, with immediate ART initiation offered at point of testing, introduction to HIV clinic staff, assistance with transport to clinic and telephone or in-person follow-up for those who missed their first appointment. For those not already on ART, 73% were linked to care and initiated on ART within 12 months [16]. Other studies, like one in Uganda, have found only modest benefit from home visits to encourage ART initiation after home-based testing (34% intervention versus 26% control; HR = 1.31; 95% CI = 0.85–2.04) [17]. In South Africa, standard of care (SoC) mobile HIV counselling and testing units were compared with point-of-care (POC) CD4 alone, POC CD4 plus up to five care facilitation counselling sessions and POC CD4 plus transport reimbursement [18]. Only POC CD4 plus care facilitation counselling sessions showed improvement over SoC (cumulative risk of verified ART initiation, 0.18 versus 0.13; *p* = 0.02) [18].

In the treatment as prevention (TasP) (ANRS 12249) cluster randomised trial in South Africa, only 30% of those newly diagnosed through home-based HIV counselling and testing linked to care within 6 months and ART initiation did not differ significantly between the immediate ART initiation and clinic-based initiation (ART was offered according to national guidelines based on CD4 count) clusters [19]. A follow-up study in the same setting assessed a program combining home-based HIV testing with SMS reminders and telephone-facilitated support to attend clinic. Timely linkage to care and ART initiation were low. ART initiation was particularly low in youth less than 30 years old (29% women and 16% men) versus older patients (41% women and 35% men) [20].

POC services for rapid ART initiation were studied in another South African population in which individuals received POC tests (CD4, tuberculosis test if symptomatic, haemoglobin, creatinine, liver function test), plus a physical exam, education, counselling and ART dispensing. ART initiation within 90 days increased (97% intervention versus 72% SoC; crude relative risk of 1.36 [1.24–1.49]), and viral suppression rates at 10 months were also higher in the intervention arm [21].

In South Africa, contracting with a non-governmental organisation to provide escorted transport from home to referral facility increased ART enrolment from 27% at baseline to 85% post intervention [22]. In Nigeria, the Community Treatment Initiative started PLHIV (who had previously refused ART) on treatment in the community. Seventy percent of this population initiated ART through the community treatment initiative, 88% (*p* < 0.0001) of those were retained in care at 6 months, and 78% of those retained in care achieved viral suppression (*p* < 0.0001) [23].

Another approach to increase ART initiation involved improved care within facilities. The START-ART strategy provided healthcare providers with education on the balance of risk and benefits of starting ART, POC CD4 testing and feedback to clinics. ART initiation within 14 days was higher in the intervention group (weighted proportion [80%] versus control [38%]) (risk ratio (RR) = 2.11; 95% CI = 2.03–2.20) [24].

Combination intervention strategies have also been assessed in a number of studies. In Mozambique, two health communication interventions (modified pre-ART counselling and SMS reminders to link to care) and three structural interventions (POC CD4 testing, accelerated ART initiation and non-cash financial incentives) were piloted to assess the feasibility and acceptability of each intervention; in a dose delivery assessment without comparison group, 53% of eligible participants (per POC CD4 testing) initiated ART within 1 month [25]. A similar combination intervention strategy (POC CD4+ testing, accelerated ART initiation, mobile phone appointment reminders, health educational packages and non-cash financial incentives) in eSwatini improved 12-month retention in care (RR = 1.48; 95% CI = 1.18–1.86; *p* = 0.002) with a non-significant increase linkage to care (RR = 1.08; 95% CI = 0.97–1.21; *p* = 0.13) and ART initiation (RR = 1.16; 95% CI = 0.96–1.40; *p* = 0.12) [26]. In a non-randomised study in Nigeria, ‘One Stop Shops’ (integrated services for HIV testing and counselling, STI treatment, clinical referrals and ART to key populations) had ART initiation rates of 25% compared to 4% in standard clinics (*p* = < 0.001) [27].

Although ART access is universal, not all individuals are ready to start immediately after diagnosis. In Kenya and South Africa, the SLATE algorithm used four screening tools (symptom self-report, medical history questionnaire, physical examination and readiness assessment) to ascertain eligibility for same-day initiation or refer for further care. Among adults presenting for HIV testing or care, but not on ART, 78% initiated ART within 28 days versus 68% in SoC (RR = 1.15) in the South African sample, while 94% initiated ART versus 89% in SoC (RR = 1.06) in the Kenyan sample. However, retention at 8 months was poor in both arms and countries [28]. Facility-based counselling to promote immediate ART initiation was also assessed in South Africa using an approach developed by Médicins Sans Frontières. A 6% increase was seen compared with SoC; however, 18-month viral suppression was no different [29].

Several studies of potentially promising interventions have not shown gains for ART initiation. No benefit was seen in a South African study involving a conditional economic incentive (39% intervention versus 45% control; adjusted odds ratio (AOR) = 0.67; 95% CI = 0.26–1.78) [30] or in a Kenyan study involving weekly text message (WelTel Retain; 82% versus control 78%) [31], or a sexual trauma coping intervention for women with a history of sexual abuse in South Africa (91% intervention versus 88% SoC) [32].

### Adolescents and Young Adults

HIV remains a leading cause of death among young people aged 15–24, and adolescent girls and young women continue to be disproportionately affected [1, 33]. Peer-delivered interventions may be particularly effective for adolescent populations based on expected age-appropriate neurodevelopment [34]. In Kenya, the Red Carpet Program interlinked community- and facility-level components to provide fast-track peer-navigated services in healthcare facilities and schools, including peer counselling and psychological support; 97% of participants were linked to care within 6 months of program implementation, compared to 57% pre intervention (*p* = < 0.001). Of those linked to care, no difference in ART initiation was seen; however, the proportion of those retained in care and taking ART at 6 months increased in a pre-post comparison (54% versus 99%; *p* = < 0.001) [35]. In South Africa, a small peer-to-peer mobile phone-based system linked newly diagnosed youth with stable youth in care, resulting in increased ART initiation (80% versus 42% in matched controls) [36]. Similarly, in a pre-post cohort design, ART uptake increased 2.5 times (95% CI = 1.6–4.0) for youth in Uganda attending peer-delivered targeted counselling and health education linked to youth-oriented sexual and reproductive health and rights facilities [37].

### Pregnant Women and Infants

Promising results have been seen in studies integrating prevention of mother-to-child transmission (PMTCT) within primary care services. When combining PMTCT with antenatal and child health services, the proportion of women on ART increased from 80 to 98% (*p* = < 0.001) in rural Tanzania [38]. This intervention included provider-initiated HIV testing, inpatient counselling and implementation of electronic medical records. Similarly, in Nigeria, women receiving PMTCT services packaged with mother and infant care were more likely to initiate ART (97% versus 39%; adjusted RR = 3.3; 95% CI = 1.4–7.8) and be retained in care [39^••^].

Interventions to link pregnant women living with HIV in the community to healthcare facilities, however, have produced mixed results. In Tanzania, community health workers (CHWs) supported linkage to healthcare facilities, provided adherence counselling, traced those LTFU and distributed a birth planning and appointment reminder tool. A non-significant 8% point increase was seen in ART initiation compared to controls [40]. Community-based ‘Expert Mothers’ (women living with HIV who recently underwent PMTCT and were on ART) providing 1:1 support, support groups and follow-up for missed appointment in Malawi resulted in higher ART initiation compared with facility-based models (86% versus 81%), although the difference was not statistically significant; retention at 12 months was also higher [41]. In the Democratic Republic of Congo, a cluster randomised trial of an intervention to promote referrals to ‘Mentor Mothers’ within a healthcare facility at their first antenatal care appointment found no difference in ART initiation compared to SoC (66% versus 63%) [42].

### Female Sex Workers

Female sex workers (FSWs) are 13 times more likely to be living with HIV than women in the general population and may benefit from tailored services [43]. Community outreach for HIV testing and immediate ART prescription at a healthcare centre in Benin resulted in high uptake of HIV testing (96%) and initial ART uptake (96%; *p* = 0.031) within a prospective observational cohort. However, retention in care was low (59% at a mean of 13 months) [44]. Building on existing community-based testing and ART counselling services in Tanzania showed significant improvement in ART initiation compared to receiving healthcare facility–based care (among the 82% attending the 6-month visit, 100% versus 72%; *p* = 0.04) [45]. Targeted combination prevention at specialist drop-in centres integrated ART or pre-exposure prophylaxis (PrEP) initiation and peer adherence support into usual care in the SAPPH-Ire trial in Zimbabwe. While ART uptake was high and detectable viral load decreased at follow-up in the intervention (30 to 16%) and control (30 to 19%) groups, a little difference was seen between groups [46]. In Uganda, although direct provision of self-test kits by peer educators to FSWs increased HIV testing, when compared to collecting self-test kits at a facility and SoC, there was no effect on ART initiation (43% versus 34% facility collection versus 45% SoC) [47].

### Tuberculosis

Interventions to identify PLHIV receiving care for concomitant TB could facilitate timely entry to the HIV care cascade and ART initiation. In South Africa, a pilot cohort study examined case managers who promoted HIV testing, followed up laboratory results, contacted patients to return for results and facilitated treatment initiation for patients being investigated for TB, resulting in 74% of eligible PLHIV initiating ART [48].

### Looking to the Future

Several ongoing studies are trialling innovative strategies, typically in the community, to engage populations at high risk of virologic failure. In South Africa, the Lotto to Link trial is using a conditional lottery incentive to engage men in HIV testing and treatment and achieve viral suppression [49] Similarly, the Zwakala Ndoda trial combines male-focused HIV testing, personalised linkage to care, case management and flexible clinic hours to encourage linkage and engagement in care for men [50]. In Zambia, the SHIELD trial aims to demonstrate the use of peer support within community-based youth clubs and tailored clinical services for young women and adolescent girls [51]. The VIBRA trial in Lesotho will build on previous evidence from Labhardt et al. [15^••^] and hopes to demonstrate the effectiveness of task-shifting same-day ART and drug refill by village health workers, with the option of adherence reminders and lab result delivery by SMS [52]. For the non-pregnant adult population in South Africa not on treatment within 3 months of HIV diagnosis, the eight-session ‘Treatment Ambassador’ program aims to address barriers to ART initiation through motivational interviewing and peer support. And, in an HIV hotspot fishing community in Uganda, ‘Health Scouts’ (CHWs) will use motivational interviewing strategies and mobile health tools to promote engagement in HIV treatment [53].

### Target Population: All PLHIV Prescribed ART

Several intervention studies have included all people living with HIV who have initiated ART, without a focus on high-risk groups such as those with sub-optimal adherence.

### General Adult Populations

SMS reminders are a low-cost strategy to support PLHIV to remember to take their ART. In South Africa, SMS reminders were sent to all PLHIV attending a rural public clinic who had provided a mobile phone number. Seventy-two percent of those with a valid phone number had at least one confirmed SMS delivery, and 81% of those remained subscribed to the reminders for the 11-month duration of the study. Exposure to the SMS reminders was significantly associated with greater daily prescription coverage (AOR = 1.23; 95% CI = 1.13–1.34; *p* < 0.001) when compared to non-exposure [54]. In rural Uganda, two different SMS reminder approaches were compared with a control group through a pilot RCT (*n* = 63). All participants were given an electronic monitoring device capable of sending cellular signals relaying participant adherence. Over 9 months, patients received either (i) a scheduled SMS reminder for the first 3 months and then SMS reminder triggered if no signal was received from the electronic monitoring device for the next 6 months or (ii) SMS triggered only if no signal received from the electronic monitoring device, plus a message to a social supporter if no signal received for > 48 h or (iii) control group receiving no SMS. Mean adherence (measured using the electronic monitoring device) was higher in the first group (91%) receiving scheduled SMS when compared to both the group receiving triggered SMS (79%) and the control group (79%) (*p* = 0.04), suggesting scheduled SMS had greater benefit [55].

Economic incentives have been used along the HIV care cascade including to encourage uptake of ART and adherence to ART. In Uganda, unconditional cash transfers were not found to improve adherence (self-report 3-day recall) when compared with a control group. The structure of unconditional cash grants was evaluated through three intervention arms: (i) unconditional cash transfer with no financial coaching, (ii) unconditional cash transfer with individual financial counselling, (iii) no financial coaching and unconditional cash transfer only at the end of study and (iv) no cash transfer, compared with (iv) HIV standard care, using a 2 × 2 factorial design [56]. However, in this study, food insecurity was not assessed which may limit the findings [56]. However, in Tanzania, conditional food and cash transfers did have a beneficial effect on ART adherence at 6 months (95% medication possession ratio) (adjusting for site, 58% (95% CI = 50, 66) SoC versus 76% (95% CI = 72, 80) intervention) particularly for food-insecure patients and those newly diagnosed [57].

A quasi-experimental pilot study in Tanzania trialled a clinic and home-based intervention using culturally appropriate images to prime patients to improve adherence. Average adherence at 6 months (medication possession ratio) was similar between the groups (90% versus 89%) [58].

In a study involving 840 PLHIV in Nigeria, attending a peer support group was significantly associated with achieving 95% or greater adherence compared to not attending a peer support group (92% versus 87%; *p* = 0.024). Adherence was measured as a percentage derived from pharmacy dispensing data and self-report missed doses. Measurements of adherence based on participant’s self-reports over 28 days are open to recall bias. Peer support groups were formal meetings of PLHIV aged 18 or over, where discussions centred on identifying barriers to adherence and problem-solving solutions [59].

### Pregnant Women

Engaging male partners in PMTCT may be an effective strategy to improve adherence to ART after ART initiation, removing barriers to adherence and facilitating adherence support. In Malawi, pregnant women who received an HIV diagnosis were asked to invite their partner for couple HIV testing and counselling (cHTC) including information on HIV prevention and adherence to ART (*n* = 200). Sixty-three percent of women attended cHTC; attending the session was not associated with increased adherence (measured by medication possession ratio). However, attending the cHTC was strongly associated with improved retention (aRR = 1.33; 95% CI = 1.12, 1.59) at 1-month ART refill [60].

### Target Population: ART Users with Sub-optimal Adherence

ART adherence patterns change often for a large number of PLHIV. The vast majority of ART users will struggle with adherence at some point in their lifetime due to issues such as non-disclosure, internalised HIV stigma, substance use and mental disorders such as depression that negatively impact adherence. Surprisingly, only six studies conducted in Africa since 2015 could be identified that targeted sub-optimal adherence. None of these studies were conducted among youth and pregnant women. Enhanced adherence counselling (EAC) is often SoC in some African countries when patients are virally unsuppressed. Yet, in many instances, what is referred to as EAC may be a repeat of what patients received at the point of initiation, and few adjustments are made in consideration of the specific needs of patients who are sub-optimally adherent. Further, the curriculum for EAC varies among countries and health systems, which may account for the differing outcomes that were obtained in the studies reviewed below.

### General Adult Populations

Two studies used single-arm designs and examined the impact of SoC adherence counselling for those who were unsuppressed on second-line ART; results differed. In Uganda, enhanced adherence counselling consisted of patients being informed of their elevated viral load and receiving three counselling sessions delivered monthly. The counsellor provided education on ART and the implications of an unsuppressed viral load (VL), reviewed the patient’s current adherence and collaboratively revised their adherence plan. Patients’ treatment buddies were also contacted when possible, although what transpired when they were contacted was not described. This approach was not found to be effective in this study, as 91% of participants remained unsuppressed with a VL > 1000 copies/mL after 3 months [61].

In contrast, in South Africa, SoC adherence counselling for those unsuppressed on second-line ART was found to be effective. The enhanced adherence counselling consisted of at least one counselling session with an experienced adherence counsellor or social worker. During this session, patients were screened for depression and alcohol and drug abuse, education was provided on ART, misconceptions were addressed and an adherence plan was developed. The patient then saw a medical officer who was experienced in treatment failure whom they continued to see until they were suppressed. This approach was reported as being effective as 64% (95% CI = 59–69) of patients were re-suppressed on second-line ART after an average of 3 months [62].

A single-arm study examined a novel intensive adherence intervention approach in patients failing second-line ART in four West African countries (Burkina Faso, Ivory Coast, Mali and Senegal). The intensive adherence intervention consisted of monthly counselling sessions where participants could choose any of the following reinforcement strategies: (i) direct observation of treatment by a relative, (ii) use of a pill organiser, (iii) weekly phone calls, (iv) daily alarm reminders, (v) daily SMS text messages, (vi) home visits, (vii) individual adherence counselling sessions, (viii) a peer self-help group and (ix) limiting non-ART drugs prescribed. This approach demonstrated promising outcomes with 67% of patients re-suppressed on second-line ART after 16 weeks [63].

The remaining three studies included a comparison or control group to examine novel approaches to improving adherence in patients failing first- or second-line ART. All reported a positive outcome in terms of self-reported adherence or achieving viral suppression. A behavioural self-regulation counselling intervention was delivered telephonically by an adherence counsellor to patients unsuppressed on first- or second-line ART in South Africa. The intervention consisted of five weekly phone calls to problem-solve patient-identified barriers to ART adherence and retention in care including internalised HIV stigma and substance use. In comparison to the control group who only received weekly check-in calls from the counsellor, participants in the intervention group reported significantly greater improvements in ART adherence (*d* = 0.69) as measured by an unnamed self-report scale [64].

In a quasi-experimental study in Ethiopia, participants, including those with suboptimal adherence, who received an economic strengthening (ES) program were 2.4 times more likely to achieve optimal adherence as measured by a visual analogue scale (95% CI = 1.699–3.435) and 5.6 times more likely to achieve optimal adherence as measured by the ACTG self-report measure (95% CI = 2.599–12.301), compared to those in the community who were not engaged in ES activities. The ES intervention involved training to build business and financial management skills; access to savings, loans and investments at village level; and opportunities to share experiences related to business skills, ART adherence, positive living and nutrition. Participants were encouraged to start businesses individually or in groups [65].

In a study using a brief active visualisation intervention in South Africa to improve adherence in first- and second-line ART failure patients, the intervention group showed a significantly greater viral suppression at follow-up (adjusted mean = 1.92; 95% CI = − 2.41 to − 1.43) in comparison with the control group (adjusted mean = − 1.24; 95% CI = − 1.76 to − 0.73) [66^••^]. Changes in colour in a perspex container of liquid when chemicals are added were used to simulate the effects of ART on viral suppression, to boost awareness off the importance of adherence [66^••^].

### Looking to the Future

Common mental disorders such as depression are highly prevalent among PLHIV in Africa and have been found to negatively impact ART adherence. Our search revealed multiple protocol papers published since 2015 on large randomised controlled trials of behavioural interventions for addressing comorbid mental disorders in HIV in Africa. One of these RCTs focuses on addressing adherence and depression in ART users with suboptimal adherence. The Ziphamandla study in South Africa is examining the effectiveness of eight sessions of nurse-administered cognitive behavioural therapy (CBT) compared to enhanced treatment as usual (ETAU) in addressing adherence and depression in ART failure patients. A cost-effectiveness analysis is included [67^••^]. The study is scheduled for completion in 2020 and is based on a pilot study conducted prior to 2015 [68]. A similar pilot study, called the TENDAI intervention, was conducted in Zimbabwe which sought to address adherence and depression in ART users with adherence difficulties using six sessions of problem-solving therapy administered by lay counsellors [69^•^]. The adherence intervention in the TENDAI study was the culturally adapted LifeSteps intervention available in the main local Zimbabwean language (Shona) [70^•^]; a large-scale RCT examining the efficacy of this intervention compared to ETAU is currently underway in Zimbabwe. If these interventions are found to be effective and cost-effective, there will be important implications for the implementation and scale-up of much-needed behavioural interventions for ART users with suboptimal adherence in primary HIV care in Africa. Integrating evidence-based mental health treatment into primary HIV care could play a critical role in achieving the UNAIDS 90-90-90 goals.

### Target Population: ART Users with Stable Adherence

#### General Adult Populations and Postpartum Women

Three randomised control trials for patients with stable adherence and a primary endpoint of viral suppression after the Treat All era were identified.

ART adherence clubs are the main intervention for patients who are stable on ART. Adherence clubs typically consist of patients aged 18 and above, on a consistent ART regimen for 1 year with two consecutive suppressed viral load results, and who do not have a medical condition requiring consultation with a doctor more than once a year [71]. In South Africa, a cluster randomised control trial was conducted to evaluate adherence clubs compared to SoC [72^•^]. In this trial, up to 30 patients met to receive prepacked medications, group adherence counselling and symptoms screening every 2 to 3 months. Viral suppression rates were similar for patients in the adherence club and SoC within 18 months. Similar findings were reported in a study comparing adherence clubs to SoC for postpartum women [73]. Similarly, an RCT compared community-based adherence clubs with clinic-based adherence clubs and found that retention of participants was better in the latter [74]. A qualitative study conducted after this RCT indicated that this finding was possibly due to less stigma, quality of service from leaders of the adherence clubs, time saved compared to attending usual care at clinics and access to additional clinic services should they be required [75].

### Looking to the Future

In Lesotho, at the time of writing, a community-based differentiated model of multi-month dispensing of ART medication is being evaluated to assess the impact of the intervention on viral suppression [76]. Postpartum adherence clubs are also being evaluated as a method of providing ART for postpartum women compared to SoC in a pragmatic RCT in South Africa [77].

### Target Population: PLHIV Initiated on Treatment Who Disengage from Care or Are Lost to Follow-up

#### Adult Populations

Drivers of poor retention in HIV care in African countries are similar to those that impact on adherence and include financial and opportunity costs of keeping regular appointments [78], weak beliefs about benefits of ART, lack of disclosure, fear of being scolded by nurses after returning following a missed appointment and common mental disorders like depression, which lower motivation and confidence in overcoming barriers [78, 79].

Prior to Treat All, influential studies, such as one in Kenya, had shown that intensive adherence counselling at and shortly after ART initiation, including both education and problem-solving around barriers to adherence, resulted in a significant impact on adherence and virologic treatment failure during an 18-month follow-up [80]. Building on this, an uncontrolled evaluation of a health systems strengthening intervention across all ages and population groups in Uganda included training and supervision of expert patients and staff in best practices for follow-up and funds for telephone follow-up [81]. Only minimal, statistically insignificant changes were seen in the proportion attending at least one ART visit by 6 months post initiation (76% versus 72%; *p* = 0.12), and there were small changes in secondary outcomes such as increase in the mean number of appointments; however, post hoc analyses showed effects to be superior for younger patients. The cost per additional patient retained in care was $47. Another intervention trialled in 14 East African sites with patients who were LTFU showed that tracing by a peer worker led to a statistically significant 22% (95% CI = 7.1–36.2) rise in return to care for patients who were able to be contacted in person and who were already known to be alive and not using services elsewhere. However, tracing at random was inefficient, leading to an increase of only 3% in return to care [82^••^]. This study advocated for face-to-face conversations that include encouragement and problem-solving.

### High-Risk Groups

Two studies targeting high-risk groups showed promising results (Table 2). Small cash transfers, conditional on attending appointments, significantly improved retention in the PMTCT cascade for newly diagnosed pregnant mothers in the Democratic Republic of Congo (DRC), with the effects of cash being stronger for the poorest women [83^••^]. However, follow-up was only to 6 weeks postpartum [83^••^]. In Tanzania, the household hunger scale was used to identify food insecurity in adults living with HIV. In this three-arm trial, food baskets and cash transfers (equivalent of $11 per month), provided for 6 months, significantly increased retention at a 6-month follow-up compared to SoC. The effect of the cash transfers persisted at 12 months of follow-up [84^••^].

**Table 2:** Studies and their characteristics on different target populations

Authors (year of publication)	Study type	Intervention type	Study period	Study country	Healthcare setting
Target population: PLHIV who know their status and are not yet initiated on treatment					
Labhardt et al. (2018) [15^••^]	Randomised controlled trial	Same-day home-based ART initiation with follow-up	February 2016–September 2017	Lesotho	Public healthcare facilities
Ayieko et al. (2019) [16]	Randomised controlled trial	Community HIV testing with rapid linkage to care and ART initiation, transport to clinic, telephone or in-person follow-up for those who missed first clinic appointment	May 2013–May 2016	Kenya and Uganda	Community-based
Ruzagira et al. (2017) [17]	Randomised controlled trial	Home-based HIV testing with post-test counselling for clinic referral	March 2015–March 2016	Uganda	Home-based
Hoffmann et al. (2017) [18]	Randomised controlled trial	POC CD4 testing with post-test counselling, care facilitation counselling and transport assistance	March 2013–October 2014	South Africa	Community-based
Iwuji et al. (2016) [19]	Randomised controlled trial	Home-based HIV testing with referral to healthcare facility for immediate ART initiation	March 2012–May 2014	South Africa	Home-based
Baisley et al. (2019) [20]	Non-randomised controlled trial	Home-based HIV testing with enhanced telephone-facilitated support	January 2017–May 2018	South Africa	Home-based
Rosen et al. (2016) [21]	Randomised controlled trial	Rapid single-visit ART initiation with POC blood tests, physical exam and ART education	May 2013–August 2014	South Africa	Public healthcare facilities
Shamu et al. (2019) [22]	Non-randomised controlled trial	Community-based HIV testing and counselling program, referral escorts to facility to expedited HIV initiation at facility	October 2015–September 2016	South Africa	Community-based
Katbi et al. (2019) [23]	Non-randomised controlled trial	Targeted community-based ART initiation with counselling and ART education pre-initiation; 4-week ART prescription then linked to a healthcare facility for continued treatment and care	October 2015–June 2016	Nigeria	Community-based
Amanyire et al. (2016) [24]	Randomised controlled trial	Training and coaching of healthcare workers regarding the risks and benefits of offering ART, POC CD4 testing, removal of mandatory multiple pre-ART initiation visits and feedback to the facilities on their ART initiation rates	April 2013–February 2015	Uganda	Public healthcare facilities
Sutton et al. (2017) [25]	Randomised controlled trial	Modified delivery of pre-ART counselling sessions, SMS reminders, POC CD4 testing, accelerated ART initiation and non-cash financial incentives	April 2013–June 2015	Mozambique	Public healthcare facilities
McNairy et al. (2017) [26]	Randomised controlled trial	POC CD4+ testing, accelerated ART initiation for treatment-eligible participants, mobile phone appointment reminders, health educational packages and non-cash financial incentives	August 2013–November 2015	eSwatini	Public healthcare facilities
Onovo et al. (2016) [27]	Non-randomised controlled trial	Community-based One-Stop Shops providing integrated services for HIV testing and counselling, STI treatment, clinical referrals and ART	June 2015–December 2015	Nigeria	Community-based
Rosen et al. (2019) [28]	Randomised controlled trial	Algorithm to ascertain eligibility for same-day initiation	March 2017–April 2018	South Africa	Public healthcare facilities
Pascoe et al. (2019) [29]	Randomised controlled trial	Fast-track treatment initiation; ART education and adherence counselling provided once before ART initiation, at initiation and at 1 month and 2 months post initiation	January 2016–December 2016	South Africa	Public healthcare facilities
Maughan-Brown et al. (2018) [30]	Randomised controlled trial	Conditional economic incentive on linkage to care and uptake of treatment following ART referral by a mobile health clinic	April 2015–May 2016	South Africa	Community-based
van der Kop et al. (2018) [31]	Non-randomised controlled trial	Weekly interactive text messages, which are followed up by telephone calls if patient indicate that they have an issue	April 2013–June 2015	Kenya	Community-based
Sikkema et al. (2018) [32]	Non-randomised controlled trial	Trauma-focused coping intervention delivered as 4 1:1 sessions and 3 group sessions, for HIV-infected women with sexual abuse histories	August 2014–October 2018	South Africa	Public healthcare facilities
Ruria et al. (2017) [35]	Non-randomised controlled trial	Fast-track peer-navigated services, peer counselling and psychosocial support at healthcare facilities	July 2015–December 2016	Kenya	Public healthcare facilities and community outreach
Hacking et al. (2019) [36]	Non-randomised controlled trial	Peer mentorship via mobile phones for newly diagnosed youth	March 2015–May 2016	South Africa	Public healthcare facilities
Vu et al. (2017) [37]	Non-randomised controlled trial	Peer-delivered education and counselling and link to SRH facilities	March 2013–December 2014	Uganda	Public healthcare facilities
Gamell et al. (2016) [38]	Non-randomised controlled trial	Integrated PMTCT clinic within reproductive and child health clinic, implementation of electronic medical records, provider-initiated HIV testing	December 2012–December 2014	Tanzania	Public healthcare facilities
Aliyu et al. (2016) [39^••^]	Randomised controlled trial	Point-of-care CD4 testing, integrated mother and infant service provision and male partner and community engagement	April 2013–March 2014	Nigeria	Public healthcare facilities
Nance et al. (2017) [40]	Randomised controlled trial	Community health workers facilitate linkage to health facilities and provide adherence counselling, loss to follow-up tracing and distribution of a birth planning tool	January 2014–March 2015	Tanzania	Community-based
Phiri et al. (2017) [41]	Randomised controlled trial	Peer support for PMTCT based in the facility or in the community providing 1:1 support and support groups, appointment reminders and HIV education	November 2013–July 2016	Malawi	Public healthcare facilities
Gill et al. (2018) [42]	Randomised controlled trial	Standard operating procedure with detailed guidance for referral to care for HIV-positive pregnant women receiving ANC services	May 2015–November 2015	Democratic Republic of Congo	Public healthcare facilities
Mboup et al. (2018) [44]	Non-randomised controlled trial	HIV testing assessment with CD4, viral load blood tests for female sex workers; immediate ART or PrEP initiation when informed of HIV test result	October 2014–December 2016	Benin	Specialist clinic
Tun et al. (2019) [45]	Non-randomised controlled trial	Community-based ART distribution program for HIV-positive female sex workers	July 2017–October 2018	Tanzania	Community-based
Cowan et al. (2018) [46]	Randomised controlled trial	Combination HIV prevention and treatment intervention package providing HIV prevention and sexual health services plus assisted referral to HIV treatment	January 2014–November 2014	Zimbabwe	Specialist public healthcare facilities and community outreach sites
Ortblad et al. (2017) [47]	Non-randomised controlled trial	HIV self-testing delivery models to increase HIV testing and linkage to care	October 2016–November 2016	Uganda	Community-based
Maraba et al. (2018) [48]	Non-randomised controlled trial	Case manager facilitated care, promoting testing, getting laboratory results, calling patients to return for results and facilitating treatment initiation	September 2014–April 2015	South Africa	Public healthcare facilities
Target population: all PLHIV prescribed ART					
Georgette et al. (2017) [54]	Non-randomised controlled trial	Weekly scheduled SMS on antiretroviral therapy adherence support	July 2013–July 2014	South Africa	Public healthcare facilities
Haberer et al. (2016) [55]	Randomised controlled trial	4 types of SMS plus real-time adherence monitoring on ART adherence: daily reminders, weekly reminders, reminders triggered after a late or missed dose (delivered to patients) and notifications triggered by sustained adherence lapses (delivered to patient-nominated social supporters)	September 2013–July 2015	Uganda	Public healthcare facilities
Mills et al. (2018) [56]	Randomised controlled trial	Unconditional cash transfers with no financial coaching, unconditional cash transfers with individual financial counselling and no financial coaching and unconditional cash transfers only at the end of study	October 2013–May 2014	Uganda	Community-based
Kadota et al. (2018)* [57]	Randomised controlled trial	Short-term cash, food assistance, nutrition assessment and counselling	December 2013–August 2016	Tanzania	Public healthcare facilities
McCoy et al. (2017) [58]	Non-randomised controlled trial	Reward of clinic attendance using stickers on a clinic-based poster and adherence reminder priming using image on clinic poster, annual calendar or pill box	May 2016–July 2016	Tanzania	Public healthcare facilities
Chime et al. (2018) [59]	Non-randomised controlled trial	Adult peer support groups addressing health-related issues and management of HIV, problem-solving around barriers to adherence	September 2016–December 2016	Nigeria	Public healthcare facilities
Wesevich et al. (2017) [60]	Randomised controlled trial	Couple HIV testing and counselling, early disclosure of HIV-positive status, partner ART reminders	March 2014–November 2014	Malawi	Antenatal clinic
Target population: those on ART with suboptimal adherence					
Birungi et al. (2020) [61]	Non-randomised controlled trial	Fast-track, enhanced adherence counselling for first-line ART failure patients, treatment buddies contacted when possible, VL repeated after 3 months	July 2012–December 2013	Uganda	NGO-funded healthcare facility and outreach sites
Fox et al. (2016) [62]	Non-randomised controlled trial	Fast-track, enhanced adherence counselling for second-line ART failure patients, continuity of care with the same medical officer, VL repeated after 2–4 months	March 2012–December 2013	South Africa	Public healthcare facilities
Eholie et al. (2019) [63]	Non-randomised controlled trial	Intensive adherence counselling with 9 optional adherence reinforcement interventions for second-line ART failure patients, VL repeated after 12 weeks	March 2013–May 2015	Burkina Faso, Cote d’Ivoire, Mali, Senegal	Public healthcare facilities
Kalichman et al. (2019) [64]	Randomised controlled trial	Behavioural self-regulation counselling for unsuppressed ART users	August 2017–October 2017	South Africa	Public healthcare facilities
Bezabih et al. (2018) [65]	Non-randomised controlled trial	Economic strengthening program	September 2014–September 2017	Ethiopia	Public healthcare facilities
Jones et al. (2019) [66^••^]	Randomised controlled trial	Active visualisation device demonstrating how a liquid changes colour to simulate changes in viral load shown to first- and second-line ART failure patients	May 2016–November 2016	South Africa	Public healthcare facilities
Target population: interventions for ART users with stable adherence					
Fox et al. (2019) [72^•^]	Randomised controlled trial	Antiretroviral adherence clubs	March 2016–March 2017	South Africa	Public healthcare facilities and community-based
Myer et al. (2017) [73]	Non-randomised controlled trial	Antiretroviral adherence clubs	September 2015–April 2016	South Africa	Community-based
Hanrahan et al. (2019) [74]	Randomised controlled trial	Adherence clubs	February 2014–February 2016	South Africa	Public healthcare facilities and community-based
Target population: PLHIV initiated on treatment who disengage from care or are lost to follow-up (LTFU)					
Boeke et al. (2018) [81]	Non-randomised controlled trial	Quality of care improvement intervention package (training lay health workers in best practices for patient follow-up and counselling, improved appointment recordkeeping, phone calls and home visits to LTFU patients, enhanced adherence counselling strategies)	September 2016–August 2017	Uganda	Public healthcare facilities
Bershetyn et al. (2017) [82^••^]	Non-randomised controlled trial	Tracing patients LTFU	January 2008–January 2010	Uganda, Kenya, Tanzania	Community clinics
Yotebieng et al. (2016) [83^••^]	Randomised controlled trial	Cash incentives to remain in care	April 2013–August 2014	Democratic Republic of Congo	Public healthcare facilities
McCoy et al. (2017)** [84^••^]	Randomised controlled trial	Short-term cash, food assistance, nutrition assessment and counselling	December 2013–August 2016	Tanzania	Public healthcare facilities

*Trial also reported in “Target population: PLHIV initiated on treatment who disengage from care or are lost to follow-up (LTFU)” [84^••^]

**Trial also reported in “Target population: all PLHIV prescribed ART” [57]

## Discussion

This scoping review describes interventions which have been tested for their effect in promoting uptake or adherence to ART in African countries in the Treat All era. We presented this evidence across five phases of the care cascade, including PLHIV who have recently learned their status, those already initiated, those who are and who are not experiencing periods of sub-optimal ART adherence and those disengaged from ART care. Most studies we found were of interventions aimed to improve ART initiation. The majority of the studies were conducted in East or Southern Africa, with South Africa and then Uganda accounting for the highest number of studies. Half (52%) of the studies presented here were RCTs.

ART initiation can be improved through a variety of mechanisms, including peer support to adolescents [36, 37]; integration of ART with antenatal and family care services [38, 39^••^]; health services strengthening, community outreach and linkage to facilities [15^••^, 23, 85]; and provision of point-of-care services [21, 24]. These interventions, however, were not universally successful, and impact varied by context and/or study methods [17, 20, 30, 31]. For general adult populations, linking PLHIV to care as they receive their diagnosis either by actively facilitating first appointments at an HIV care facility or by providing fast and simplified treatment services such as point-of-care blood tests with rapid ART initiation has been shown to improve uptake of ART [15^••^, 21, 23]. Future research should explore how to implement and scale successful programs in real-world settings.

For PLHIV who have initiated treatment, more resources and more research should be directed to those negotiating challenges in adhering at various points through their treatment trajectory. For example, intensive counselling or economic support interventions for key populations and high-risk groups, such as young postpartum women, when they are at increased risk of non-adherence because of changes in circumstances and major life events. Evidence from high-income countries has shown brief interventions for PLHIV with comorbid mental disorders are effective in increasing adherence to ART [86]. In Africa more treatment studies of common mental disorders in PLHIV are needed, to test whether this will improve adherence and viral suppression. Such research must make use of culturally validated tools for mental disorders. Effective strategies should be developed to identify PLHIV at risk of non-adherence to ART or who have sub-optimal adherence who may benefit from targeted interventions, such as culturally adapted adherence interventions based on cognitive behavioural principles which extend beyond standard adherence counselling [70^•^].

Tailoring interventions to cultures which differ from those for whom the intervention was originally developed typically follows the following sequence: information gathering, preliminary adaptation design and testing, and adaptation refinement [87]. The range of changes to be made may include the language of delivery, the people and resources available, the content, ways to enhance acceptability and metaphors to bring local meaning. If resources for the adaptation process are limited, a lot can be achieved through bringing all the critically relevant stakeholders, including those with lived experience, together over a short number of workshops, developing a theory of change for the intervention [88] and using the information to make the preliminary changes. With more resources, which may be available through seed grants from funders, a process such as the ADAPT-ITT model [89] can be followed which begins with literature review and robust qualitative research to inform the preliminary adaptation design [90], followed by formal assessment of feasibility and acceptability of the adapted intervention [69^•^]. Interventions to be adapted should be derived from those with an evidence base and be viewed by local stakeholders as meaningful in the local context with the potential to be incorporated into existing services and spaces.

Adherence clubs are commonly used in African countries for stable patients. This model should be scaled up, as it ensures patients are able to receive their care quickly and are not burdened with long waiting queues and frequent clinic visits [75]. Importantly, viral suppression for those attending Adherence Clubs was no different than SoC, indicating the safety (or non-inferiority) of this differentiated care approach. However, innovative interventions are needed for young people who are stable on ART, reducing the need for frequent clinic visits which impact on education, domestic responsibilities and social activities and can thus be a barrier to ART adherence.

For PLHIV initiated on ART but have disengaged from care, providing cash incentives shows distinct promise in improving retention in HIV care, for perinatal women living with HIV and those who are food insecure [83^••^, 84^••^]. Amounts needed to make a difference are likely to be small, covering transport costs of getting to clinics and possibly making a minimal contribution to basic food items [81]. Tracing those LTFU is widely practised in African countries, but the evidence we found suggests this should be performed in a targeted way, prioritising those who can be seen face-to-face by a peer worker who has been trained to be encouraging and use problem-solving approaches [81, 82^••^]. Current evidence is lacking on cost-effectiveness of comprehensive health systems strengthening interventions to improve retention in care.

Unfortunately, much work remains to strengthen the evidence base on adherence interventions in African nations. The quality and reporting of primary outcome(s), intervention content and participant population was particularly poor in some studies identified through our search. For instance, many studies used blanket descriptions of *adherence counselling* that did not include the theory or methods used, and objective adherence data was lacking. Many of the studies provided only weak evidence as they lacked a control group. More robust evidence is needed for the cost-effectiveness of novel scalable interventions, and studies should include a control condition, be adequately powered to detect an effect and use biological outcomes like viral load (an objective measure of sufficient adherence, not necessarily of high and consistent adherence) and dried blood spot for detection of ART drug concentrations. These outcomes may be achievable to collect in low- and middle-income countries through making strong equitable partnerships with those overseeing routine viral load monitoring (such as ministries of health and PEPFAR-supported facilities) and through building local capacity, for instance through supporting transport of specimens and cost-sharing staff and reagents. Where biological outcomes are not feasible, pharmacy refill or self-report adherence can be collected, but measures used should be those with demonstrated reliability and which have been validated against viral load [91]. Electronic adherence monitors are another informative option when feasible. This increased rigour will require additional capacity, such as functional viral load test machines at public healthcare facilities with effective procedures for returning results. Current ongoing trials focus on innovative strategies to engage PLHIV who are less likely to engage in care or are at high risk of non-adherence. These include community strategies to engage men [49, 50], peer support and tailored services for adolescent girls and young women [52] and community-based ART dispensing and task-shifted counselling techniques based on CBT for those with common mental disorders [67^••^, 69^•^].

A limitation of this review is the lack of a formal quality assessment for the articles reviewed, which may lead to bias. We were constrained by the sheer number of papers but have distinguished RCTs from non-randomised and non-controlled studies. Another limitation was our focus on English language papers, which may have excluded studies from Francophone and Lusophone African countries [92].

## Conclusions

Enormous progress has been made in the Test and Treat era to ensure all PLHIV are able to access ART soon after diagnosis. However, uptake of ART and continuous adherence to ART are fluid across the life course. Many African nations have policies in place that detail strategies to support PLHIV to adhere to ART to maintain viral suppression. Investment is needed in differentiated approaches for those with sub-optimal adherence, for those who have disengaged from care and for young adults. Robust research is needed across sub-Saharan Africa to test scalable interventions—research which includes appropriate control groups, economic evaluation and objective measures of adherence. Measures of adherence for future trials should include long-term biological outcomes such as sustained viral suppression and dried blood spot (DBS) testing for the detection of ART presence. A sustained commitment to improving adherence to ART is vital to improve the quality of life for people living with HIV and will be needed to end the HIV epidemic.

## Electronic Supplementary Material

ESM 1 (DOCX 35 kb)
