# Supplementary material for: A Cascade of Interventions to Promote Adherence to Antiretroviral Therapy in African Countries
Source: Curr HIV/AIDS Rep. 2020 Aug 10;17(5):529–46. doi: 10.1007/s11904-020-00511-4 (PMC7497365; doi:10.1007/s11904-020-00511-4)
Supplement: Supplementary file 1 — (DOCX 35 kb) [file 11904_2020_511_MOESM1_ESM.docx]

Supplementary Material 1:

Search Strategy December 2019

**Research Goal:** This scoping review discusses strategies to improve adherence for five target populations along five components of the HIV treatment cascade: (1) people living with HIV (PLHIV) who know their status and are not yet initiated on antiretroviral therapy (ART), (2) ART users with sub-optimal adherence, (3) ART users with stable adherence, and (4) PLHIV initiated on treatment who disengage from care for periods of time or are lost to follow up (LTFU).

# Methods of the review:

1. Electronic searches of:

PubMed/MEDLINE

Cochrane Central Register of Controlled Trials (CENTRAL) Web of Science

EMBASE

1. Studies that matched with search string will be downloaded into RAYYAN* and screened by title for relevance to eliminate titles/topics that were irrelevant. Abstracts will be reviewed for references of unknown relevance.
2. Review abstracts for relevant articles
3. Full copies of selected articles will be reviewed to determine relevance
4. Data extraction of effect sizes

**Rayyan is a free, online application developed by Qatar Computing Research Institute designed to expedite the initial screening of abstracts and titles.*

Mourad Ouzzani, Hossam Hammady, Zbys Fedorowicz, and Ahmed Elmagarmid. Rayyan — a web and mobile app for systematic reviews. *Systematic Reviews* (2016) 5:210, DOI: 10.1186/s13643-016-0384-4. URL: <https://systematicreviewsjournal.biomedcentral.com/articles/10.1186/s13643-016-0384-4>

**Search 1: PLHIV who know their status and are not yet initiated on ART**

1. PubMed Search Strings: MeSH & free text combos (December 2019)

|  | | **Search Terms** | | **Format used** | |
| --- | --- | --- | --- | --- | --- |
| HIV | | HIV OR  Human Immunodeficiency Virus OR HIV infection OR Acquired Immunodeficiency Syndrome OR AIDS OR HIV infections | | (((((((((((((((HIV[MeSH Terms]) OR human immunodeficiency virus[MeSH Terms]) OR hiv infection[MeSH Terms]) OR acquired immunodeficiency syndrome[MeSH Terms]) OR AIDS[MeSH Terms]) OR HIV) OR human immunodeficiency virus) OR AIDS) OR HIV infection) OR hiv infections[MeSH Terms]) OR HIV infections))) | |
| SSA | | Africa OR Africa South of the Sahara | | (((((((((Africa[MeSH Terms]) OR Africa south of the sahara[MeSH Terms]) OR Sub-Saharan Africa) OR Subsaharan Africa) OR Africa, Sub-Saharan) OR LMIC) OR (low and middle-income countries)) OR (low and middle-income countries)) OR resource-limited)) | |
| Linkage | | Linkage OR Linkage to Care OR re-engagement OR re-engage OR  engagement or enrollment OR enroll OR return to care OR engage OR link OR link to care OR attrition OR lost to follow-up OR lost to follow up OR retention | | ((((((((((((((linkage) OR  linkage to care) OR re-engagement) OR engagement) OR  enrollment) OR enroll) OR return to care) OR engage) OR link) OR link to care) OR attrition) OR lost to follow-up) OR lost to follow up) OR retention) OR re-engage | |
| Trial design | | randomized controlled trial [pt]  controlled clinical trial [pt] | | ((((((((((((randomized controlled trial [pt]) OR controlled clinical trial [pt]) OR randomized [tiab]) OR placebo [tiab]) OR clinical trials as topic [mesh: noexp]) OR randomly [tiab]) OR trial [ti]) OR comparison group [tiab]) OR standard of care [tiab]) OR intervention[tiab])) | |
|  | | randomized [tiab] | |  |  |
|  | | placebo [tiab] | |  |  |
|  | | clinical trials as topic [mesh: noexp] | |  |  |
|  | | randomly [tiab] | |  |  |
|  | | trial [ti] | |  |  |
|  | Comparison group [tiab] Standard of care [tiab] intervention[tiab]  #1 OR #2 OR #3 OR #4 OR #5 OR #6  OR #7 OR #8 OR #9 OR #10 | |  | |  |
| Combined |  | | (((((((((((((((HIV[MeSH  Terms]) OR human immunodeficiency virus[MeSH Terms]) OR hiv infection[MeSH Terms]) OR acquired immunodeficiency syndrome[MeSH Terms]) OR AIDS[MeSH Terms])  OR HIV) OR human immunodeficiency virus) OR AIDS) OR HIV  infection) OR hiv infections[MeSH Terms]) OR HIV infections))) AND (((((((((Africa[MeSH Terms]) OR africa south of the sahara[MeSH Terms]) OR Sub-Saharan Africa) OR Subsaharan Africa) OR Africa, Sub-Saharan) OR LMIC) OR (low and middle-income countries))  OR (low and  middle-income countries)) OR resource-limited)) AND ((((((((((((((((linkage)  OR linkage to care) OR re-engagement) OR engagement) OR  enrollment) OR enroll) OR return to care) OR engage) OR link) OR link to care) OR attrition) OR lost to follow-up) OR lost to follow up) OR retention) OR re-engage))) AND ((((((((((((randomized controlled trial [pt]) OR controlled clinical trial [pt]) OR randomized [tiab]) OR placebo [tiab]) OR clinical trials as topic [mesh: noexp]) OR randomly [tiab]) OR trial [ti]) OR comparison group [tiab]) OR standard of care [tiab])  OR intervention[tiab])) NOT ((animals [mh] NOT humans [mh]))) | |  |
| Filters activated: Publication date from 2014/01/01 to 2019/12/31 | | | | |  |
| **Total hits: 764** | | | | |  |

-------------------------------------------------------------------------------------------------------------------------------------------

1. Web of Science: free text used (December 2019)

| **Research Aspect** | **Search Terms** | **Format used** |
| --- | --- | --- |
| HIV | HIV OR human immunodeficiency virus OR hiv infection OR acquired immunodeficiency syndrome OR AIDS OR HIV infections (restricted to ... | TS=(HIV) OR TS=(human immunodeficiency virus) OR TS=(hiv infection) OR TS=(acquired immunodeficiency syndrome) OR TS=(AIDS) |
| Sub-Saharan Africa | Africa OR Sub-Saharan Africa OR Subsaharan Africa OR Africa Sub-Saharan OR Africa south of the Sahara OR LMIC OR resource-limited OR low and middle-income | TS=(Africa) OR TS=(africa south of the sahara) OR TS=(Sub-Saharan Africa) OR TS=( Subsaharan Africa) OR TS=(Africa Sub-Saharan) OR TS=(LMIC OR low and middle-income countries) OR TS=(low and middle-income countries) OR TS=(resource-limited) OR TS=(resource-constrained) |
| Adherence | Linkage OR Linkage to Care OR re-engagement OR  re-engage OR engagement or enrollment OR enroll OR return to care OR engage OR link OR link to care OR attrition OR lost to follow-up OR lost to follow up OR retention OR loss to follow-up OR loss to follow up OR ART initiation | TS=(adherence) OR TS=(adherent) OR TS=(re-engagement) OR TS=(re-engage) OR TS=(engagement) OR TS=(engage) OR TS=(return to care) OR TS=(attrition) OR TS=(lost to follow-up) OR TS=(lost to follow up) OR TS=(retention) OR TS=(Compliance) OR TS=(adherence) OR TS=(ART adherence) OR TS=(sub-optimal adherence) |
| RCT/quasi | Clinical trial OR intervention OR random* OR controlled trial OR placebo OR standard of care OR control OR comparison group | TS=(randomized) controlled trial OR TS=(controlled clinical trial) OR TS=(randomized) OR TS=(placebo) OR TS=(clinical trials) OR TS=(randomly) OR TS=(trial) OR TS=(comparison group) OR TS=(standard of care ) OR TS=(intervention) |
| Combined >2014 |  | #4 AND #3 AND #2 AND  #1  Refined by: PUBLICATION YEARS: (2020 OR 2019 OR 2018 OR 2017 OR 2016  OR 2015 OR 2014 )  Timespan: All Years. |
| **Total hits: 1,998** | | |

-------------------------------------------------------------------------------------------------------------------------------------------

1. CENTRAL (Cochrane)- free text (December 2019)

| **Research Aspect** | **Search Terms** | **Format used** |
| --- | --- | --- |
| HIV | HIV OR human immunodeficiency virus OR hiv infection OR acquired immunodeficiency syndrome OR AIDS OR HIV infections | (HIV) OR (human immunodeficiency virus) OR (hiv infection*) OR (acquired immunodeficiency syndrome) OR (AIDS) |
| Sub-Saharan Africa | Africa OR Sub-Saharan Africa OR Subsaharan Africa OR Africa Sub-Saharan OR Africa south of the Sahara OR LMIC | (Africa) OR  (sub-Saharan Africa) OR (Africa south of the Sahara) OR (LMIC) OR |
| Linkage to Care | Linkage OR Linkage to Care OR re-engagement OR  re-engage OR engagement or enrollment OR enroll OR return to care OR engage OR link OR link to care OR attrition OR lost to follow-up OR lost to follow up OR retention OR loss to follow-up OR loss to follow up OR ART initiation OR low and middle-income OR resource-limited | (linkage) OR  (re-engage*) OR ("lost to follow-up") OR (initiation) OR (retention) OR ("low and middle-income") |
| RCT/quasi | N/A | N/A |
| Combined | #1 AND #2 AND #3 | #4 - #1 and #2 and #3 (in trials) |
| **Total Hits: 432** | | |

-------------------------------------------------------------------------------------------------------------------------------------------

1. EMBASE (December 2019)

| **Research Aspect** | **Search Terms** | **Format used** |
| --- | --- | --- |
| HIV | HIV OR human immunodeficiency virus OR hiv infection OR acquired immunodeficiency syndrome OR AIDS OR HIV infections | hiv OR (human AND immunodeficiency AND virus) OR (hiv AND infection) OR (acquired AND immunodeficiency AND syndrome) OR aids OR (hiv AND infections) |
| Sub-Saharan Africa | Africa OR Sub-Saharan Africa OR Subsaharan Africa OR Africa Sub-Saharan OR Africa south of the Sahara OR LMIC OR low and middle-income OR resource-limited | africa OR 'africa south of the sahara' OR ('sub saharan' AND africa) OR (subsaharan AND africa) OR lmic OR (low AND 'middle income') OR 'resource limited' |
| Linkage to Care | Linkage OR Linkage to Care OR re-engagement OR  re-engage OR engagement or enrollment OR enroll OR return to care OR engage OR link OR link to care OR attrition OR lost to follow-up OR lost to follow up OR retention OR loss to follow-up OR loss to follow up OR ART initiation | linkage OR 'linkage to care' OR 're engagement' OR 're engage' OR engagement OR enrollment OR enroll OR (return AND to AND care) OR engage OR link OR (link AND to AND care) OR attrition OR (lost AND to AND 'follow up') OR (lost AND to AND follow AND up) OR retention OR (loss AND to AND 'follow up') OR (loss AND to AND follow AND up) OR (art AND initiation) |
| RCT/quasi | Clinical trial OR intervention OR random OR controlled trial OR placebo OR standard of care OR comparison group | 'clinical trial' OR 'controlled clinical trial' OR 'intervention study' OR intervention OR 'randomized controlled trial' OR 'controlled study' OR placebo OR (standard AND of AND care) OR (comparison AND group) |
| Combined | #1 AND #2 AND #3 AND #4 | #1 AND #2 AND #3 AND  #4 |
| Combined, limit by years | > 2014 | #5 AND (2014:py OR 2015:py OR 2016:py OR 2017:py OR 2018:py OR 2019:py OR 2020:py) |
| **Total Hits: 2,345** | | |

**Search 2: People living with HIV prescribed ART**

1. PubMed Search Strings: MeSH & free text combos (January 2020)

|  | **Search Terms** | **Format used** |
| --- | --- | --- |
| HIV | HIV OR Human Immunodeficiency Virus OR HIV infection OR Acquired Immunodeficiency Syndrome OR AIDS OR HIV infections | HIV[MeSH Terms] OR human immunodeficiency virus[MeSH Terms] OR hiv infection[MeSH Terms] OR acquired immunodeficiency syndrome[MeSH Terms] OR AIDS[MeSH Terms] OR HIV OR human immunodeficiency virus OR AIDS OR HIV infection OR hiv infections[MeSH Terms] OR HIV infections ))) |
| SSA | Africa OR Africa South of the Sahara | (((((((((Africa[MeSH Terms] OR africa south of the sahara [MeSH Terms] OR Sub-Saharan Africa OR Subsaharan Africa OR Africa, Sub-Saharan OR LMIC OR low and middle-income countries OR low and middle-income countries OR resource-limited OR resource-constrained)) |
| Adherence/Re-Engagement | Adherence OR Adherent OR Re-Engagement OR Re-Engage OR Engagement OR Engage OR Return to Care OR Lost to follow-up OR Lost to Follow Up OR Compliance OR adherence OR ART Adherence OR Sub-Optimal Adherence | ((((((((((adherence [tiab] OR adherent [tiab] OR re-engagement [tiab] OR re-engage [tiab] OR engagement [tiab] OR engage [tiab] OR return to care [tiab] OR attrition [tiab] OR lost to follow-up [tiab] OR lost to follow up [tiab] OR retention [tiab] OR Compliance [tiab] OR adherence [tiab] OR ART adherence [tiab] OR sub-optimal adherence [tiab])) |
| Trial design | randomized controlled trial [pt]  controlled clinical trial [pt] | ((((((((((((randomized controlled trial [pt]) OR controlled clinical trial [pt]) OR randomized [tiab]) OR placebo [tiab]) OR clinical trials as topic [mesh: noexp]) OR randomly [tiab]) OR trial [ti]) OR comparison group [tiab]) OR standard of care [tiab]) OR intervention[tiab])) |
|  | randomized [tiab] |  |
|  | placebo [tiab] |  |
|  | clinical trials as topic [mesh: noexp]  randomly [tiab |  |
|  | randomly [tiab] |  |
|  | trial [ti]  Comparison group [tiab] Standard of care [tiab] intervention[tiab]  #1 OR #2 OR #3 OR #4 OR #5 OR #6  OR #7 OR #8 OR #9 OR #10 |  |
| Combined |  | HIV[MeSH Terms] OR human immunodeficiency virus[MeSH Terms] OR hiv infection[MeSH Terms] OR acquired immunodeficiency syndrome[MeSH Terms] OR AIDS[MeSH Terms] OR HIV OR human immunodeficiency virus OR AIDS OR HIV infection OR hiv infections[MeSH Terms] OR HIV infections ))) AND (((((((((Africa[MeSH Terms] OR africa south of the sahara [MeSH Terms] OR Sub-Saharan Africa OR Subsaharan Africa OR Africa, Sub-Saharan OR LMIC OR low and middle-income countries OR low and middle-income countries OR resource-limited OR resource-constrained)) AND ((((((((((adherence [tiab] OR adherent [tiab] OR re-engagement [tiab] OR re-engage [tiab] OR engagement [tiab] OR engage [tiab] OR return to care [tiab] OR attrition [tiab] OR lost to follow-up [tiab] OR lost to follow up [tiab] OR retention [tiab] OR Compliance [tiab] OR adherence [tiab] OR ART adherence [tiab] OR sub-optimal adherence [tiab])) AND ((((((((((((randomized controlled trial [pt]) OR controlled clinical trial [pt]) OR randomized [tiab]) OR placebo [tiab]) OR clinical trials as topic [mesh: noexp]) OR randomly [tiab]) OR trial [ti]) OR comparison group [tiab]) OR standard of care [tiab]) OR intervention[tiab])) |
| **Total Hits: 883** | | |

-------------------------------------------------------------------------------------------------------------------------------------------

1. Web of Science: free text used (January 2020)

| **Research Aspect** | **Search Terms** | **Format used** |
| --- | --- | --- |
| HIV | HIV OR human immunodeficiency virus OR hiv infection OR acquired immunodeficiency syndrome OR AIDS OR HIV infections (restricted to ... | TS=(HIV) OR TS=(human immunodeficiency virus) OR TS=(hiv infection) OR TS=(acquired immunodeficiency syndrome) OR TS=(AIDS) |
| SSA | Africa OR Sub-Saharan Africa OR Subsaharan Africa OR Africa Sub-Saharan OR Africa south of the Sahara OR LMIC OR resource-limited OR low and middle-income | TS=(Africa) OR TS=(africa south of the sahara) OR TS=(Sub-Saharan Africa) OR TS=( Subsaharan Africa) OR TS=(Africa Sub-Saharan) OR TS=(LMIC OR low and middle-income countries) OR TS=(low and middle-income countries) OR TS=(resource-limited) OR TS=(resource-constrained) |
| Adherence/Re-Engagement | Adherence OR Adherent OR Re-Engagement | TS=(adherence) OR TS=(adherent) OR TS=(re-engagement) OR TS=(re-engage) OR TS=(engagement) OR TS=(engage) OR TS=(return to care) OR TS=(attrition) OR TS=(lost to follow-up) OR TS=(lost to follow up) OR TS=(retention) OR TS=(Compliance) OR TS=(adherence) OR TS=(ART adherence) OR TS=(sub-optimal adherence) |
| Combined |  | #4 AND #3 AND #2 AND  #1 |
| Combined >2014 |  | #4 AND #3 AND #2 AND  #1  Refined by: PUBLICATION YEARS: (2020 OR 2019 OR 2018 OR 2017 OR 2016  OR 2015 OR 2014 )  Timespan: All years. |
| **Total hits: 2,030** | | |

-------------------------------------------------------------------------------------------------------------------------------------------

1. CENTRAL (Cochrane)- free text (January 2020)

| **Research Aspect** | **Search Terms** | **Format used** |
| --- | --- | --- |
| HIV | HIV OR human immunodeficiency virus OR hiv infection OR acquired immunodeficiency syndrome OR AIDS | (HIV) OR (human immunodeficiency virus) OR (hiv infection*) OR (acquired immunodeficiency syndrome) OR (AIDS) |
| Sub-Saharan Africa | Africa OR Sub-Saharan Africa OR Subsaharan Africa OR Africa Sub-Saharan OR Africa south of the Sahara OR LMIC | (Africa) OR (africa south of the sahara) OR (Sub-Saharan Africa) OR (LMIC) |
| Adherence/Re-Engagement | Linkage OR Linkage to Care OR re-engagement OR  re-engage OR engagement or enrollment OR enroll OR return to care OR engage OR link OR link to care OR attrition OR lost to follow-up OR lost to follow up OR retention OR loss to follow-up OR loss to follow up OR ART initiation OR low and middle-income OR resource-limited | (linkage) OR  (re-engage*) OR ("lost to follow-up") OR (initiation) OR (retention) OR ("low and middle-income") |
| RCT/quasi | N/A | N/A |
| Combined | #1 AND #2 AND #3 | #4 - #1 and #2 and #3 (in trials) |
| **Total Hits: 438** | | |

-------------------------------------------------------------------------------------------------------------------------------------------

1. EMBASE (January 2020)

| **Research Aspect** | **Search Terms** | **Format used** |
| --- | --- | --- |
| HIV | HIV OR human immunodeficiency virus OR hiv infection OR acquired immunodeficiency syndrome OR AIDS OR HIV infections | hiv OR (human AND immunodeficiency AND virus) OR (hiv AND infection) OR (acquired AND immunodeficiency AND syndrome) OR aids OR (hiv AND infections) |
| Sub-Saharan Africa | Africa OR Sub-Saharan Africa OR Subsaharan Africa OR Africa Sub-Saharan OR Africa south of the Sahara OR LMIC OR low and middle-income OR resource-limited | africa OR 'africa south of the sahara' OR ('sub saharan' AND africa) OR (subsaharan AND africa) OR lmic OR (low AND 'middle income') OR 'resource limited' |
| Adherence/Re-Engagement | Adherence OR Adherent OR Re-Engagement OR Re-Engage OR Engagement OR Engage OR Return to Care OR Attrition OR Lost To Follow Up OR Retention OR Compliance OR ART Adherence OR Sub-Optimal Adherence | adherence OR adherent OR re-engagement OR re-engage OR engagement OR engage OR (return AND to AND care) OR attrition OR (lost AND to AND follow AND up) OR retention OR compliance OR (art AND adherence) OR (sub-optimal AND adherence) |
| RCT/quasi | Clinical trial OR intervention OR random OR controlled trial OR placebo OR standard of care OR comparison group | 'clinical trial' OR 'controlled clinical trial' OR 'intervention study' OR intervention OR 'randomized controlled trial' OR 'controlled study' OR placebo OR (standard AND of AND care) OR (comparison AND group) |
| Combined | #1 AND #2 AND #3 AND #4 | #1 AND #2 AND #3 AND  #4 |
| Combined, limit by years | > 2014 | #5 AND (2014:py OR 2015:py OR 2016:py OR 2017:py OR 2018:py OR 2019:py OR 2020:py) |
| **Total Hits: 1,555** | | |
